# Plasma Inflammatory Cytokines Are Elevated in ALS

**DOI:** 10.3389/fneur.2020.552295

**Published:** 2020-11-13

**Authors:** Rosanna Tortelli, Chiara Zecca, Marco Piccininni, Sara Benmahamed, Maria Teresa Dell'Abate, Maria Rosaria Barulli, Rosa Capozzo, Petronilla Battista, Giancarlo Logroscino

**Affiliations:** ^1^Center for Neurodegenerative Diseases and the Aging Brain, University of Bari “Aldo Moro” - A.O. Pia Fond “Card. G. Panico” Hospital, Lecce, Italy; ^2^UCL Queen Square Institute of Neurology, University College London, London, United Kingdom; ^3^Institute of Public Health, Charité—Universitätsmedizin Berlin, Berlin, Germany; ^4^UMR_S 1094, Tropical Neuroepidemiology, Institute of Neuroepidemiology and Tropical Neurology, University Limoges, CNRS FR 3503 GEIST, Limoges, France; ^5^Istituti Clinici Scientifici Maugeri IRCCS, Institute of Bari, Pavia, Italy; ^6^Global Brain Health Institute (GBHI), University of California, San Francisco, San Francisco, CA, United States; ^7^Department of Basic Medical Science and Sense Organs, University of Bari “Aldo Moro,” Bari, Italy

**Keywords:** cytokines, plasma, ALS, biomarker, inflammation

## Abstract

Amyotrophic lateral sclerosis (ALS) is a progressive neurodegenerative disease which leads to death in a median time of 2–3 years. Inflammation has been claimed important to the ALS pathogenesis, but its role is still not well-characterized. In the present study, a panel of five cytokines (IL-2, IL-6, IL-10, IFN-gamma, and TNF-alpha) measured in plasma has been investigated in ALS. These biomarkers of inflammation were measured in a population-based cohort of 79 patients with ALS and 79 age- and sex-matched healthy controls using the Bio-Plex technology (Bio-Rad). All the five cytokines were significantly increased in plasma samples of patients compared with controls (*p* < 0.0001), with IL-6 having the highest median concentration (10.11 pg/ml) in the ALS group. Furthermore, IL-6 was the plasma cytokine with the highest discrimination ability between patients and controls according to the receiver operating characteristic analysis (area under the curve = 0.93). At a cut-off point of 5.71 pg/ml, it was able to classify patients and controls with 91% of sensitivity and 87% of specificity. In the ALS group, plasma IL-6 concentration correlated with demographic (age: rs = 0.25, *p* = 0.025) and clinical (revised ALS Functional Rating Scale at evaluation: rs = −0.32, *p* = 0.007; Manual Muscle Testing: rs = −0.33, *p* = 0.004; progression: rs = 0.29, *p* = 0.0395) parameters. In line with previous studies, our results confirm that inflammatory cytokines are elevated in ALS, supporting a possible role of inflammation in disease mechanism and progression. However, the precise role of inflammation in ALS needs to be further investigated on larger samples and with more mechanistic studies.

## Introduction

Amyotrophic lateral sclerosis (ALS) is a neurodegenerative disease that involves primarily motor neurons in the brain and spinal cord leading to progressive muscular atrophy, paralysis, speech and swallowing disturbances, and respiratory dysfunction (1). Death is caused by respiratory failure and occurs typically 3–5 years after diagnosis, although in some cases survival is longer than two decades (1). From a genetic point of view, the majority of ALS cases are sporadic (sALS). However, approximately 10% of cases can be considered familial (fALS) because a first- or second-degree relative with the disease can be identified.

It is now largely recognized that ALS is a complex disorder, characterized by phenotypic, genetic, and pathophysiological heterogeneity (2). The biological mechanisms causing the progressive neuronal death and the phenotypic manifestations of the disease are still not completely understood, and involve different pathways, including abnormal RNA metabolism (3), altered mitochondrial function and regulation of oxidative balance, modulation of neuronal excitability, axonal transport, control of the inflammatory response, and protein folding and degradation, in the disease pathogenesis (4).

Several evidence from human and animal studies have proved the involvement of both inflammation (local, innate immunity) and immune response (peripheral adaptive response) in ALS. First, the association between ALS and autoimmune diseases has already been described (5) and recently, TBK1 (TANK-binding kinase 1), a gene involved in inflammatory response and innate immunity, has been linked to the ALS/FTD spectrum (6, 7). Second, microglia and astroglia proliferation and activation have been mentioned as prominent histological features in the spinal cord and motor cortex of ALS patients both in postmortem and *in vivo* PET studies (8–11). Furthermore, infiltrates of macrophages and T lymphocytes have been reported in both the central and peripheral nervous system of ALS patients and in animal models (12, 13), as well as other central and peripheral immune abnormalities. Elevated levels of pro-inflammatory chemokines and cytokines and other markers of inflammation such as ferritin and C-reactive protein have been showed in plasma and cerebrospinal fluid (CSF) of patients with ALS (14, 15), sometimes with conflicting results. A recent meta-analysis involving 25 studies on peripheral cytokines levels in ALS confirmed that tumor necrosis factor-alpha (TNF-alpha), TNF receptor 1 (TNFR1), interleukin 6 (IL-6), IL-1β, IL-8, and vascular endothelial growth factor (VEGF) measured in blood were significantly elevated in ALS cases compared with controls (16). However, the role of central and peripheral cytokines in ALS, either beneficial or detrimental, and their relationship with the stage of the disease, as well as their utility as diagnostic and prognostic biomarkers in ALS, need to be further elucidated.

Currently, the ALS diagnosis is primarily based on medical history, clinical examination, and electrophysiological and imaging findings. ALS is generally diagnosed too late, when the neuronal degeneration has already spread out and significant symptoms and disability have occurred. There is, therefore, a wide urge of sensitive and specific biomarkers to help in earlier diagnosis and phenotypic characterization.

In this context, this study aims to further investigate, in a population-based setting, the role of plasma levels of inflammatory biomarkers in the diagnosis and phenotypic characterization of ALS.

## Materials and Methods

### Study Population

A sub-sample of incident sporadic ALS cases, diagnosed between 2012 and 2013 according to El Escorial criteria (17), was retrospectively selected from a population-based registry in Puglia (SLAP), Southern Italy.

The registry was established in 1997 and the surveillance began on January 1, 1998. Methodological details of the registry were published elsewhere (18). One age- (±3 years) and sex-matched healthy control was enrolled for each case. Control subjects were selected, between 2012 and 2013, from the general population of the region through reporting by their general practitioners, who were called to report subjects in good health, without acute and chronic illnesses.

Inclusion criteria for cases were (1) availability of plasma samples and (2) presence of an age- and sex-matched healthy control. Exclusion criteria for cases were: (1) familial ALS; (2) a concomitant diagnosis of any type of dementia including frontotemporal dementia; (3) history of major psychiatric disorders; (4) therapy with antidepressant (i.e., SSRIs), unless used for pain and sialorrhea in ALS patients; (5) history of chronic systemic inflammatory diseases; (6) history of localized inflammatory processes over the last month; (7) use of antibiotics or anti-inflammatory drugs over the last month. Exclusion criteria for controls were (1) presence of a first- and/or a second-degree relative with a diagnosis of ALS, frontotemporal dementia, or Alzheimer's disease; (2) history of major psychiatric disorders; (3) therapy with antidepressant (i.e., SSRIs); (4) presence of major cardiac, renal, liver, or other systemic diseases; (5) history of chronic systemic inflammatory diseases; (6) history of localized inflammatory processes over the last month; and (7) use of antibiotics or anti-inflammatory drugs over the last month.

### Procedures of Assessment

Clinical assessment of cases and coltrols was described elsewhere (19). In brief, detailed family and personal history was collected for each case and control through a structured questionnaire. All cases underwent a neurological examination by ALS-expert neurologists who were, at that time, unaware of any laboratory results. Particular attention were dedicated at the identification of upper motor neuron and lower motor neuron signs and in their distribution over several body regions. The spreading pattern of the disease was delineated using (1) the time to diffusion (TTD), as the time of symptom spreading from the onset region to a second one; and (2) the time to generalization (TTG), as the time of symptom spreading from the spinal or bulbar localization to both. These two clinical variables were based mostly on the personal history of the patient or on a direct neurological examination at baseline in a minority of cases (mostly when the neurologist detected signs, as fasciculation or spasticity in one region referred as not affected by the patient); in this case, TTD and/or TTG were considered to be present at the time of enrollment. Functional status of all cases at entry was assessed using the revised ALS Functional Rating Scale (ALSFRS-R) (20) and the Manual Muscle Testing (MMT) (21). A rate of progression was calculated as the difference between the ALSFRS-R score at diagnosis and the ALSFRS-R score at the evaluation, divided by the number of months between the diagnosis and the evaluation.

To better define, at the time of enrollment, the disease burden as measured by clinical involvement and significant feeding or respiratory failure, King's staging system was used. Five stages were considered: stage 1, symptom onset (involvement of first region); stage 2A, diagnosis; stage 2B, involvement of second region; stage 3, involvement of third region; stage 4A, need for gastrostomy; stage 4B, need for non-invasive ventilation.

Six clinical phenotypes at diagnosis were considered: ALS with prevalence of upper motor neuron signs, ALS with prevalence of lower motor neuron signs, “flail arm,” “flail leg,” bulbar ALS, and classical ALS. The phenotype “bulbar ALS” was used only for those cases who had not developed any spinal involvement in the first 6 months from the onset of symptoms and who had developed pyramidal signs before or after 6-months from symptoms' onset (2). Respiratory status of patients was assessed by forced vital capacity (FVC) and sniff nasal inspiratory pressure (SNIP). Body measures (i.e., height and weight) were collected and used to calculate body mass index (BMI) (kg/m^2^) as expression of nutritional status.

Each patient was screened for the following genes: SOD1, TDP43, FUS, VEGF, and C9orf72 and underwent a blood sample collection for biomarker analysis.

To verify if the patients were alive or dead, a medical administrative database of Apulia region (Edotto) in which mortality data are registered was consulted at the end of the study (July 1, 2020). We assumed that no ALS patient migrated outside the region during the study period.

For patients and controls, a written informed consent has been obtained. The study was approved by the Institutional Review Board of the “Azienda Sanitaria Locale, Lecce.”

All methods were carried out in accordance with relevant guidelines and regulations.

### Sample Collection and Storage

At the time of clinical assessment, each subject underwent a blood sample collection. The samples were stored in a biobank and revaluated later for the purposes of the study. In detail, venous blood was drawn by venipuncture from all cases and controls in the morning after an overnight fast. Plasma samples were collected in EDTA vacutainers, which were immediately centrifuged for 15 min at ~2,000×*g* at room temperature. After centrifugation, plasma was removed, aliquoted into polypropylene tubes, and stored at −80°C until biochemical analyses, without being thawed and re-frozen. Samples were thawed at room temperature before analysis. All samples were considered eligible for analysis, being free from plasma indexes (hemoglobin, bilirubin, and triglycerides) that could have interfered with the analytical method.

### Analytical Method

The cytokine analysis was performed using the Bio-Plex technology (Bio-Rad Laboratories). This analytical platform consists of a fluorescent bead-based technology combined with a sandwich immunoassay. This allows individual and multiplex analysis of up to 100 different analytes in a single microtiter well. A panel of five cytokines, namely human IL-2, IL-6, IL-10, IFN-gamma, and TNF-alpha, was measured for this study. The assay (Bio-Rad Laboratories, Hercules, CA, USA) was carried out at Bioclarma srl, Torino, Italy. In brief, serum samples were diluted 1:4 in sample diluents, and analyzed in 96-well microplates, accordingly to manufacturer instructions. The content of each well was then pumped into the Bio-Plex 100 System array reader (Bio-Rad Laboratories), to quantify each specific reaction based on the bead color and on the intensity of the fluorescent signal. The obtained data were finally processed using the Bio-Plex Manager software (version 6.1) according to a five-parametric curve fitting and converted in picograms per milliliter.

### Statistical Analysis

Patients' characteristics are reported as median, range, and interquartile range (IQR) for quantitative variables, and frequencies and percentages for categorical variables.

The comparison of quantitative variable distribution between ALS patients and matched healthy controls was performed with a paired Wilcoxon signed-rank test. The choice of this non-parametric procedure was driven by the typical skewed and non-normal distribution of concentration variables, as confirmed by Shapiro–Wilk normality test.

For each biomarker, the “out-of-range” (OOR) was evaluated. OOR-low results were imputed as the lower limit of the calibration curve divided by 2.

Receiver operating characteristic (ROC) analysis was conducted to measure the ability of each biomarker to discriminate between ALS patients and healthy subjects. The ROC curve for each biomarker's concentration was used to detect the optimal cut-off considering both sensitivity and specificity [point closest to (0, 1) corner in the ROC plane was selected as “optimal”].

Discrimination ability of each biomarker was quantified computing the area under the curve (AUC). The AUC, sensitivity, and specificity were calculated along with their 95% bootstrapped CI, considering 2,000 sampling replications.

Logistic regression models were used to study the ability to discriminate between ALS and controls for more biomarkers jointly.

AUC corrected for optimism were also computed for internal validation using the bootstrap method with 150 sampling replications.

Among ALS patients, univariate associations between biomarker's concentration and demographic or clinical variables were measured by Spearman's rank correlation for quantitative variables, and Kruskal–Wallis one-way ANOVA or Mann–Whitney *U* test for qualitative ones. Cox proportional-hazards regression models were used to assess the association between biomarker's levels and survival.

Only complete case analyses were conducted and *p*-values lower than 0.05 were considered as statistically significant. All analyses were performed using RStudio version 1.1.456 and R 3.6.0.

## Results

### Demographic and Clinical Characteristics of the Enrolled Population

We enrolled 79 cases and the same number of age- and sex-matched healthy controls. A descriptive analysis of the demographic and clinical characteristics of the study population is showed in Table 1.

**Table 1:** Demographic and clinical characteristic of enrolled subjects.

	**ALS**	**Controls**
Number of enrolled patients	79	79
Age (years)		
median (range)	67 (51–85)	66 (50–87)
(IQR)	(61.50–71.00)	(62.50–70.50)
Gender *n* (%)		
Male	40 (50.6%)	40 (50.6%)
Female	39 (49.4%)	39 (49.4%)
BMI (kg/m^2^); (*n* = 73)		
Median (range)	24.24 (15.62–35.32)	
(IQR)	(22.04–26.70)	
El Escorial diagnostic categories at first evaluation *n* (%)		
Definite	11 (13.9%)	
Probable	34 (43.1%)	
Possible	25 (31.6%)	
Suspected	9 (11.4%)	
Disease onset *n* (%)		
Bulbar	26 (33.3%)	
Spinal	52 (66.7%)	
Disease duration from symptoms onset (months); (*n* = 77)		
Median (range)	21.37 (3.50–137.47)	
(IQR)	(15.20–32.60)	
ODI (months); (*n* = 77)		
Median (range)	11.30 (1.30–133.03)	
(IQR)	(6.97–22.33)	
TTD (months); (*n* = 61)		
Median (range)	10.10 (0.00–94.33)	
(IQR)	4.10–17.30	
TTG (months); (*n* = 60)		
Median (range)	11.23 (1.00–119.70)	
(IQR)	6.08–20.26	
ALSFRS-R at diagnosis; (*n* = 57)		
Median (range)	40 (24–47)	
(IQR)	(35.00–43.00)	
ALSFRS-R at evaluation; (*n* = 68)		
Median (range)	33 (2–47)	
(IQR)	(24.00–39.00)	
King's staging *n* (%)		
2A	5 (6.4%)	
2B	8 (10.1%)	
3	51 (64.6%)	
4A	7 (8.9%)	
4B	8 (10.1%)	
MMT; (*n* = 70)		
Median (range)	8.90 (0.00–10.00)	
(IQR)	(7.70–9.30)	
FVC (%); (*n* = 53)		
median (range)	89.10 (1.61–134.10)	
(IQR)	(64.00–100.00)	
SNIP; (*n* = 36)		
Median (range)	56.00 (14.00–134.00)	
(IQR)	(37.75–77.25)	
Progression rate; (n = 49)		
Median (range)	0.51 (−0.72 to 14.07)	
(IQR)	(0.00–1.34)	
Phenotype *n* (%)		
ALS with prevalence of upper motor neuron signs	8 (10.8%)	
ALS with prevalence of lower motor neuron signs	19 (25.7%)	
Flail arm	6 (8.1%)	
Flail leg	1 (1.4%)	
Bulbar	9 (12.2%)	
Classical ALS	31 (41.9%)	

*ALS, amyotrophic lateral sclerosis; ODI, onset-diagnosis interval; TTD, time to diffusion; TTG, time to generalization; ALSFRS-R, Amyotrophic Lateral Sclerosis Functional Rating scale Revised; MMT, Manual Muscle Test; FVC, forced vital capacity; SNIP, Sniff Nasal Inspiratory Pressure; IQR, interquartile range*.

The median age at evaluation in ALS patients was 67 years (range: 51–85), whereas in healthy controls it was 66 years (range: 50–87). In each group, 40 (50.6%) subjects were male and 39 (49.4%) were female. Based on El Escorial Criteria, we enrolled 9 (11.4%) suspected, 25 (31.6%) possible, 34 (43.1%) probable, and 11 (13.9%) definite ALS at the time of evaluation. The median disease duration from symptom onset to evaluation was 21.37 months (range: 3.50–137.47); the median onset–diagnosis interval was 11.30 months (range: 1.30–133.03); the median TTD was 10.10 months (range: 0.00–94.33), while the median TTG was 11.23 months (range: 1.00–119.70). The median ALSFRS-R at diagnosis was 40 (range: 24–47), while the median of ALSFRS-R at evaluation was 33 (range: 2–47). Twenty-six (33.3%) cases presented a bulbar onset of the disease. Regarding the clinical phenotypes at diagnosis, 8 (10.8%) cases were ALS with prevalence of upper motor neuron signs, 19 (25.7%) ALS with prevalence of lower motor neuron signs, 6 (8.1%) flail arm, 1 (1.4%) flail leg, 9 (12.2%) bulbar ALS, and 31 (41.9%) were classical ALS. At time of enrollment, 5 (6.4%) of ALS patients were classified as King's stage 2A, 8 (10.1%) as stage 2B, 51 (64.6%) as stage 3, 7 (8.9%) as stage 4A, and 8 (10.1%) as stage 4B. No patients were classified as King's stage 1.

No mutations of the explored genes were found for any of the subjects.

### Cytokines: Group Comparisons and Discrimination Ability

Descriptive statistics of plasma levels of each cytokine in cases and controls are reported in Table 2.

**Table 2:** Biomarker comparison.

	**ALS**	**Controls**	***P-*value**
IL-2 (pg/ml)	3.22 (0.12–497.10)	0.17 (0.17–11.57)	<0.0001
IL-6 (pg/ml)	10.11 (4.22–615.46)	1.16 (0.07–40.54)	<0.0001
IL-10 (pg/ml)	1.81 (0.16–2424.92)	0.16 (0.10–109.99)	<0.0001
TNF-alpha (pg/ml)	2.97 (0.45–111.03)	0.45 (0.05–93.61)	<0.0001
IFN-gamma (pg/ml)	1.13 (0.10–231.17)	0.11 (0.04–73.53)	<0.0001

*The results are expressed as median and range of concentration*.

*ALS, amyotrophic lateral sclerosis; IL-2, interleukin 2; IL-6, interleukin 6; IL-10, interleukin 10; TNF-alpha, tumor necrosis factor alpha; INF-gamma, interferon gamma*.

**Figure 1:**
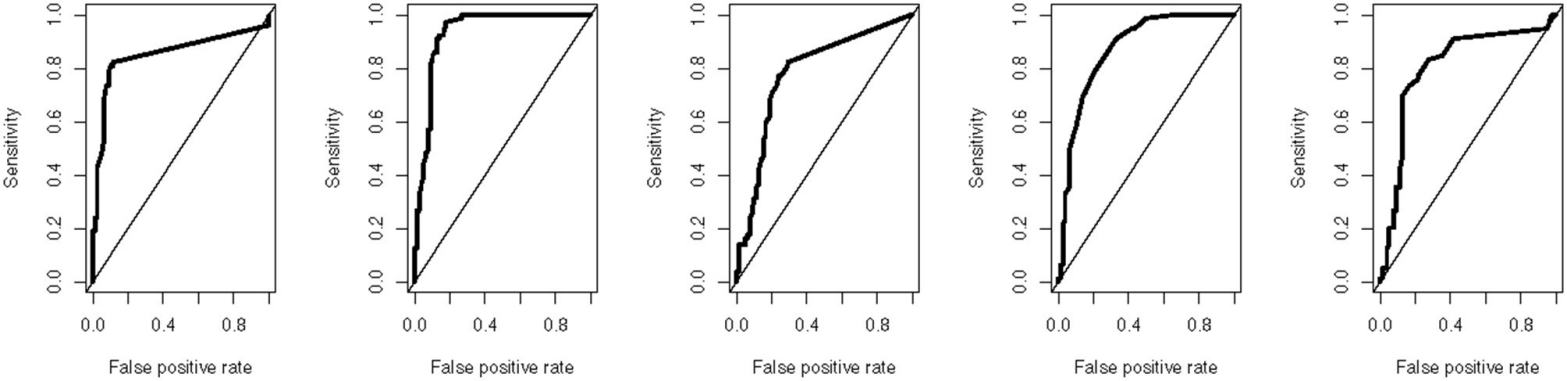
Biomarker levels in ALS patients and controls. The figure shows the plasma levels (pg/ml) of the five cytokines under investigation in the study groups, ALS (blue) and controls (red). The horizontal lines represent the median of the distribution. The y-axes are truncated to 620 pg/ml to facilitate the visualization.

**Figure 2:**
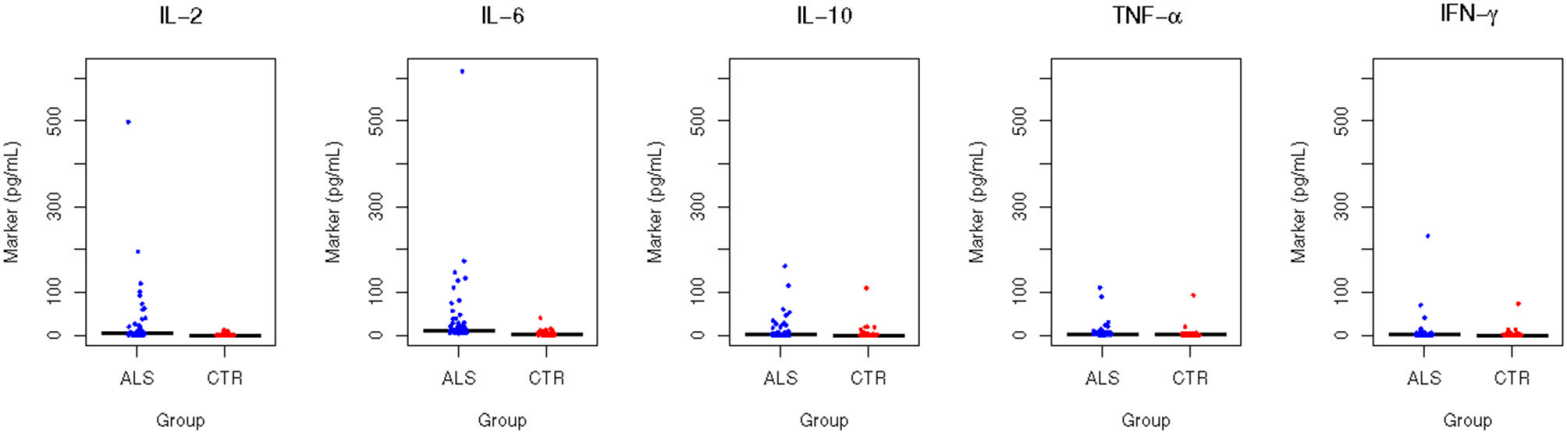
Area under the curve (AUC). The figure shows the receiver operating characteristic (ROC) curves for the five cytokines under investigation.

All the measured plasma biomarkers of inflammation were significantly higher in cases compared to controls (*p* < 0.0001). Among the measured biomarkers, IL-6 had the highest median plasma concentration in the ALS group (10.11 pg/ml, about 8.7 times the concentration in the controls), whereas IFN-gamma has the lowest median plasma concentration in the ALS group (1.13 pg/ml, about 10.3 times the concentration in the controls) (Figure 1). Using ROC analyses to investigate the diagnostic accuracy of each biomarker, IL-6 resulted to be the best candidate, showing the highest discrimination ability. Indeed, its AUC was 0.933 (0.887–0.970) (Supplementary Table 1, Figure 2).

For this cytokine, a concentration equal or higher to 5.71 pg/ml was found to be the best rule for distinguishing between cases and controls, with a sensitivity of 0.911 (95% CI 0.8481–0.9747) and a specificity of 0.873 (95% CI 0.798–0.937). Furthermore, we calculated the discrimination ability (between cases and controls) of a model encompassing the best three biomarkers (IL-6, IL-2, and TNF-alpha) as covariates and we found an AUC of 0.947 (0.938 after correction for optimism). This value was substantially unchanged by the inclusion of the other two biomarkers (5-covariates model AUC = 0.948 and 0.932 after correction for optimism).

### Cytokines: Correlation With Clinical Measures

Restricting the analysis to cases only, we evaluated the correlation between inflammatory biomarkers and clinical parameters. The correlation plot is reported in Supplementary Figure 1. IL-6 correlated positively with age (rs = 0.25; *p* = 0.025) and negatively with ALSFRS-R at evaluation (rs = −0.32; *p* = 0.007) and MMT (rs = −0.33; *p* = 0.004). All the biomarkers positively correlated between each other, except the IFN-gamma that had no statistically significant correlation with any variable under investigation (Supplementary Figure 1). IL-6 (rs = 0.30; *p* = 0.040), IL-10 (rs = 0.35; *p* = 0.014), and TNF-alpha (rs = 0.36; *p* = 0.011) had a statistically significant correlation with progression rate. There was no statistically significant correlation between cytokine levels and the stages of the King's system.

IL-10 had a statistically significant positive correlation with FVC (%) (rs = 0.37; *p* = 0.006) and negative correlation with BMI (rs = −0.32; *p* = 0.005). There was no statistically significant correlation with SNIP. Moreover, no statistically significant correlation was found between IL-6 and respiratory function tests.

Furthermore, we performed a subgroup analysis to compare biomarker levels among different patient categories according to site of onset and clinical phenotype at diagnosis. There were no statistically significant differences in biomarker levels between patients with bulbar or spinal onset of the diseases, neither between the different ALS phenotypes (data not shown).

Finally, survival analysis showed that all biomarkers had no statistically significant association with mortality (hazard ratio point estimates ranging from 0.999 to 1.001).

## Discussion

In line with previous studies (15, 16, 22–24), we confirmed an increased level of inflammatory cytokines in plasma of patients with ALS, compared with healthy controls matched for age and sex, underlying a possible role of inflammation in ALS. However, if inflammation and immune response are important triggers in disease pathogenesis and progression or a secondary response to neuronal damage, and if they are either detrimental or beneficial is still a matter of debate. Neuroinflammation and peripheral immune response have been also described in other neurodegenerative diseases, such as Parkinson's disease (PD) (25) and Alzheimer's disease (AD) (26, 27), suggesting that activation of inflammatory pathways can be a common secondary feature of a general process of neurodegeneration. At the same time, definite sub-characteristics of immune response seem to be specific for ALS. A recent meta-analysis on peripheral blood inflammatory cytokine levels in ALS, conducted by Hu and co-workers, (16) showed that although levels of TNF-alpha, IL-6, and IL-1β are not specific for ALS because they were found elevated also in AD and PD (28, 29), IL-8 and vascular endothelial growth factor (VEGF) are specifically elevated in ALS and not in other neurodegenerative diseases (16) This may suggest that inflammatory response in ALS is characterized by immune cell activation and signaling networks for cell interactions specific for ALS, but still not well-characterized (30) For example, inflammation in SOD1 mice consists mainly of inflammosomes, activated by NOD-like receptors after contact with detrimental signals. This is followed by the activation of caspase 1 and IL-1, causing a more severe disease in SOD1 mice (31). Furthermore, Meissner and co-workers showed that SOD1 mice deficient in caspase 1 or in IL-1 or treated with IL-1 receptor antagonists, have an increased lifespan (32). However, it is still not known if this mechanism applies to patients with ALS and no SOD1 mutations. However, it is unlikely that the alteration of the immune system is a primary cause of disease. Naor and colleagues demonstrated that SOD1 mice deficient in B cells still get the disease (33) and SOD1 mice depleted of microglia have the same disease as those with normal microglia, as showed by Gowing and colleagues (34). Furthermore, inflammatory response, as confirmed by our results, is independent of ALS phenotypes and not useful to distinguish between different phenotypical manifestations of the disease.

Neuroinflammation seems to rather have a role in disease progression. The CSF levels of several chemokines have been found to correlate with measures of disease progression (24), as long as the CSF levels of IL-8 have been demonstrated to increase with physical disability (35). According to these results, our study highlighted a correlation between plasma levels of IL-6, IL-10, TNF-alpha, and disease progression, and a correlation of IL-6 with measures of functional disability and muscle weakness. IL-6 plays a central role in ALS and is involved in several ALS pathways. First, IL-6 increases fatty acid oxidation–induced lipolysis and glucose metabolism, and regulates the crosstalk between muscles and adipose tissue (36). It is hypothesized that in patients with ALS, the observed systemic immune response may be correlated with the hypermetabolic state and with the lipid deregulation described in ALS (37). Second, IL-6 may act as a neurotrophic factor in response to neuronal degeneration in ALS and may also act as a marker for overall humoral immune response (23, 38). Moreover, IL-6 is secreted by T lymphocytes and macrophages as a pro-inflammatory mediator (39). Evidence suggests that T lymphocytes accumulate in regions of motor neuron loss; accumulation of T cells can in turn lead to further recruitment and activation of microglia, and cytotoxic mediators produced by these cells may be detrimental to motor neurons (35). This may explain the relationship between higher levels of IL-6 and disease severity and progression. A correlation between IL-6 level and respiratory function has been described in a previous study in which higher IL-6 levels were associated with impeding respiratory insufficiency (40). Our results showed a correlation between IL-10, but not IL-6, and respiratory function tests.

Furthermore, in our study, IL-6 resulted to have the highest diagnostic accuracy among the studied biomarkers in distinguished between patients and healthy controls. In ALS, as in other neurodegenerative diseases, there is an urgent need of sensitive, reliable, and easy-to-obtain diagnostic, prognostic, and disease-progression biomarkers, as well as biomarkers of therapeutic response. Peripheral blood inflammatory cytokines cannot be considered a diagnostic marker for ALS, as long as they are increased in other neurodegenerative and neuro-inflammatory diseases. However, because they are easy to measure, they may be of particular interest especially because they may give an important contribution in the identification of novel disease pathways and of specific subgroups of patients. This is fundamental for patient stratification in clinical trial design and may help to further elucidate disease pathogenesis and to test new therapeutic targets faster. More than 10 RCTs using anti-inflammatory drugs or monoclonal antibodies targeting inflammatory molecules have been run in ALS over the past 3 years and some of them are still ongoing (41). IL-6 has also been investigated as a therapeutic target in ALS and a phase II clinical trial with an IL-6 blocker is still ongoing (15). From this point of view, inflammatory markers can be useful as biomarkers of therapeutic response and of disease progression.

Several strengths can be identified in the present study: (1) the enrollment of most of cases at early-intermediate stage of disease (over 80% of our population was King's stage 3 or below), which could better reflect the initial phase of neuroinflammation; (2) the use of plasma samples, more accessible than CSF for long-term and repeated immune monitoring of ALS and more appropriate to investigate systemic alterations linked to neurodegeneration. On the other hand, CSF is much more informative in the field of biomarker discovery because it is the most proximal bio-fluid to the ALS-induced neurodegeneration; (3) the use of Luminex technology, a promising technique for neurodegenerative disorders, also in clinical practice to analyze multiple plasma molecules at the same time.

However, several limitations must also be considered: (1) the use of a convenient sample of cases and controls with the same demographic characteristics (matching) likely threatens the representativeness of both groups; (2) the use of a group of healthy volunteers as controls, instead of more informative populations (i.e., ALS mimic syndromes or other neurological disorders). Further validation studies on “clinical relevant” populations (also including ALS mimic syndromes) rather than separately selected cases and controls are required to investigate a possible role of cytokines as an aid for ALS diagnosis (42); (3) the retrospective study design; (4) the collection of samples at a single time point. A longitudinal study with collection of samples at different time points to investigate the over-time trajectories of these factors is hardly recommended to further investigate the role of these analytes as biomarkers of disease progression; (5) the sensitivity (detection limit) of the used technique, although higher compared with classic ELISA tests, is still not able to catch minimal concentrations of the molecule of interest. In fact, especially in the control group, several values were “out-of-range” (OOR), but we cannot be sure whether there was absolutely no analyte in those samples. A more sensitive methodology may improve the diagnostic accuracy of the proposed biomarkers.

To sum up, the results of our study confirm the activation of inflammation and immune response at least in a subgroup of patients with ALS. In line with previous studies, plasma cytokines were found to be elevated in patients with ALS compared with healthy controls and were associated with clinical markers of disease severity. Further studies with a “clinical relevant population” (42) and with a longitudinal design are needed to investigate a possible role of some inflammatory cytokines as a diagnostic, prognostic, or disease progression biomarkers and to better clarify the role of inflammation in ALS.

## Data Availability Statement

The raw data supporting the conclusions of this article will be made available by the authors, without undue reservation.

## Ethics Statement

The studies involving human participants were reviewed and approved by Institutional Review Board of the Azienda Sanitaria Locale, Lecce. The patients/participants provided their written informed consent to participate in this study.

## Author Contributions

RT and GL conceived the study. CZ and SB conducted the experiment(s). CZ, SB, and MD collected data. MP analyzed the data. RT and CZ interpreted the results and wrote the first draft of the article. All authors contributed to substantial article revision, read, and approved the submitted version.

## Conflict of Interest

The authors declare that the research was conducted in the absence of any commercial or financial relationships that could be construed as a potential conflict of interest.

## Supplementary Material

Supplementary Figure 1 Univariate correlation between biomarkers and clinical characteristic of ALS patients. The figure describes the correlation between biomarker concentrations, clinical parameters, and demographic variables of ALS patients. The green squares indicate no significant correlation (*p* > 0.05); blue circles indicate positive correlation, whereas red circles indicate negative correlation; bigger dimension and more intense color indicate a higher absolute value of the correlation (see legend on the right).

Supplementary Table 1 ROC (Receiver Operating Characteristic) analysis.
