# Supplementary figures and images for: Plasma Inflammatory Cytokines Are Elevated in ALS
Source: Front Neurol. 2020 Nov 13;11:552295. doi: 10.3389/fneur.2020.552295 (PMC7691268; doi:10.3389/fneur.2020.552295)

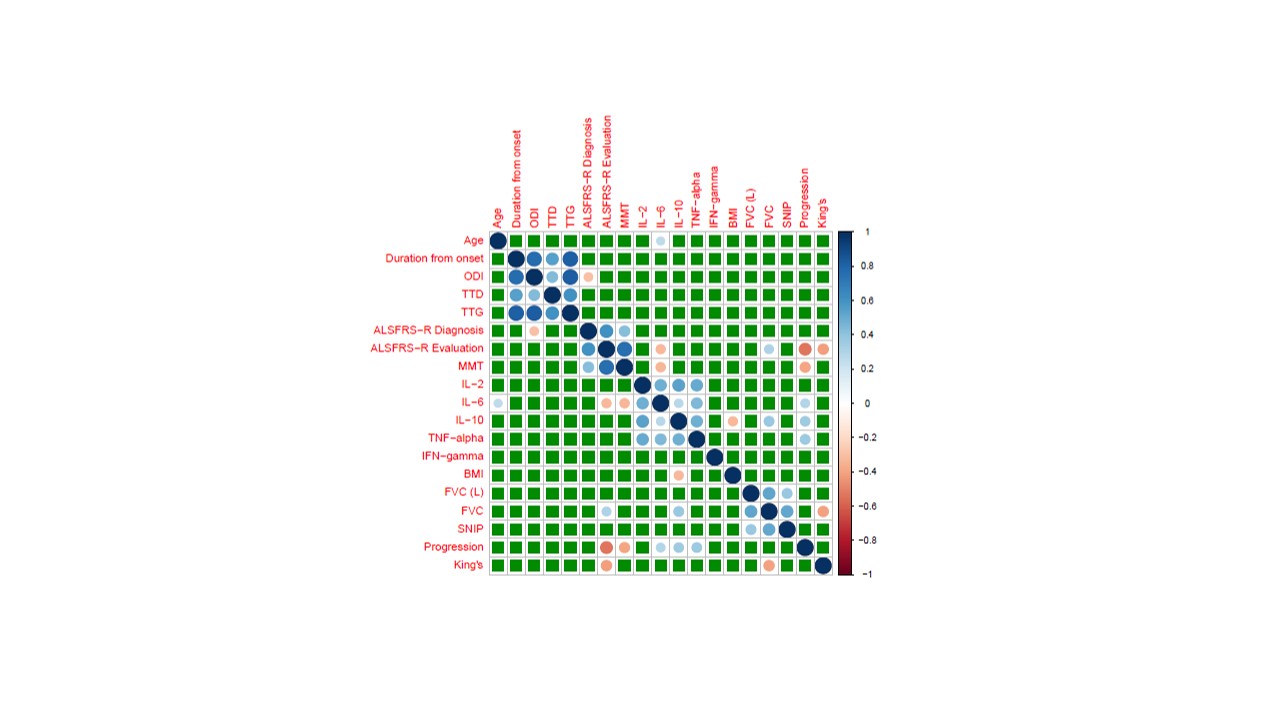

Supplement: Supplementary Figure 1 — Univariate correlation between biomarkers and clinical characteristic of ALS patients. The figure describes the correlation between biomarker concentrations, clinical parameters, and demographic variables of ALS patients. The green squares indicate no significant correlation (p > 0.05); blue circles indicate positive correlation, whereas red circles indicate negative correlation; bigger dimension and more intense color indicate a higher absolute value of the correlation (see legend on the right). [file Image_1.JPEG]
