# Supplementary material for: Plasma Inflammatory Cytokines Are Elevated in ALS
Source: Front Neurol. 2020 Nov 13;11:552295. doi: 10.3389/fneur.2020.552295 (PMC7691268; doi:10.3389/fneur.2020.552295)
Supplement: Supplementary Table 1 — ROC (Receiver Operating Characteristic) analysis. [file Table_1.DOCX]

**Supplementary Table 1: ROC (Receiver Operating Characteristic) analysis**

| Biomarkers | Cutt-off  (pg/ml) | Sensitivity  (CI 95%) | Specificity  (CI 95%) | AUC  (CI 95%) |
| --- | --- | --- | --- | --- |
| IL2 | 0.28 | 0.823  (0.73-0.90) | 0.886  (0.81-0.95) | 0.853  (0.79-0.91) |
| IL6 | 5.71 | 0.911  (0.85-0.97) | 0.873  (0.80-0.94) | 0.933  (0.89-0.97) |
| IL10 | 0.66 | 0.772  (0.68-0.86) | 0.760  (0.66-0.85) | 0.783  (0.71-0.85) |
| TNF-alpha | 2.12 | 0.785  (0.70-0.87) | 0.798  (0.71-0.89) | 0.872  (0.81-0.93) |
| IFN-gamma | 0.76 | 0.734  (0.63-0.82) | 0.835  (0.75-0.91) | 0.806  (0.73-0.88) |

CI: confidence interval; AUC: Area under the curve; IL2: Interleukin2; IL6: Interleukin6; IL10: Interleukin10;TNF-alpha: Tumor Necrosis Factor alpha; INF-gamma: Interferon gamma
